# Revisiting the roles of cAMP signalling in the progression of prostate cancer

**DOI:** 10.1042/BCJ20230297

**Published:** 2023-10-13

**Authors:** Emma C. Parsons, Ralf Hoffmann, George S. Baillie

**Affiliations:** 1 School of Cancer Sciences, Wolfson Wohl Cancer Research Centre, University of Glasgow, Bearsden, Glasgow G61 1QH, U.K.; 2 Oncology, Philips Research Eindhoven, High Tech Campus 34, 5656 AE Eindhoven, The Netherlands; 3 School of Cardiovascular & Metabolic Health, University of Glasgow, University Avenue, Glasgow G12 8QQ, U.K.

**Keywords:** androgens, cAMP, prostate cancer

## Abstract

Prostate cancer is one of the most common cancers in men and one of the top causes of death in men worldwide. Development and function of both normal prostate cells and early-stage prostate cancer cells are dependent on the cross-talk between androgen signalling systems and a variety of other transduction pathways which drive differentiation of these cells towards castration-resistance. One such signalling pathway is the ubiquitous cAMP signalling axis which functions to activate spatially restricted pools of cAMP effectors such as protein kinase A (PKA). The importance of both PKA and cAMP in the development of prostate cancer, and their interactions with the androgen receptor, were the focus of a review by Merkle and Hoffmann in 2010. In this updated review, we revisit this topic with analysis of current PKA-related prostate cancer literature and introduce novel information on the relevance of another cAMP effector, the exchange protein directly activated by cAMP (EPAC).

## Introduction

Prostate cancer is the second most common cancer diagnosis in men with an estimated 1.4 million new cases diagnosed worldwide in 2020, accounting for 14.1% of male cancer diagnoses [1]. The incidence of prostate cancer is known to increase with age, with incidence and aging-adjusted rates expected to continue to increase in most countries to 2030 [2]. Androgens are a group of hormones that are vital for the normal development and function of prostatic tissue. Initially, prostate cancer cells are dependent on androgens for growth and thus androgen deprivation therapies (ADT) have historically been the standard first line of treatment for local and metastatic hormone-sensitive prostate cancers. However, tumours may progress and grow at castrate levels of androgens, known as castration-resistant prostate cancer (CRPC). Attempts to improve treatment outcomes have led to the development of other therapies including anti-androgen, chemotherapy and radiotherapy, and combinations of these are becoming the new standard treatment for prostate cancer [3,4]. In the United States, the five-year survival rate for local or regional prostate cancer (84% of diagnoses) is over 99%, however for metastatic prostate cancer the five-year survival rate is 31% [5]. Five-year survival rates also vary with age at diagnosis and ethnicity [6]. The STOPCAP M1 systematic review and meta-analysis (*n *= 2261) aimed to assess the clinical benefit of ADT and docetaxel combination therapy and found that there was an increase in overall survival (*P *< 0.0001) as well as progression-free survival (*P *< 0.0001) and failure-free survival (*P *< 0.0001), with five-year survival improving by 9–11% [7]. This clinical benefit was strongly associated with increasing clinical T stage (*P *= 0.0019) and higher volume of metastases (*P *= 0.020). However, in patients with low volume metachronous disease there was no clinical benefit of treating with docetaxel indicating that this combination therapy is most suitable for patients with high-volume hormone-sensitive metastatic disease. Hence, there is a clear unmet need for new therapies to treat aggressive late-stage castrate-resistant prostate cancer as well as more research required on which cancer subtypes may be treated most successfully with which therapies.

Development and progression of prostate cancer has been linked to a complicated array of interacting signal transduction cascades including androgen receptor (AR) signalling, cyclic AMP (cAMP)/protein kinase A (PKA) signalling and Wnt signalling [8,9]. Most early-stage prostate cancers are androgen-dependent and so the AR is critical in driving the development of prostate cancer. AR-regulated genes include prostate-specific antigen (PSA) and prostate-specific membrane antigen (PSMA), which are commonly used biomarkers for prostate cancer. The physiological outcomes driven by cAMP signalling are mediated by two intracellular cAMP effector proteins, classic cAMP-dependent PKA [10] and the more recently discovered exchange protein directly activated by cAMP (EPAC) [11,12]. Abnormal cAMP/PKA signalling has been found in numerous cancer types, in addition to prostate cancer. cAMP and its effector PKA act to coordinate signalling cascades which are implicated in cancer cell growth and involved in cross-talk with AR signalling. This cross-talk is particularly relevant in the progression of prostate cancer from the androgen-dependent form to the castration-resistant form. This review will provide an updated snapshot on cAMP/PKA signalling in prostate cancer, including that relating to cross-talk with the AR signalling pathway.

## cAMP and PKA signalling

cAMP was first discovered over 60 years ago and is perhaps the most researched second messenger, implicated in a diverse range of cellular processes including growth, differentiation and gene transcription [13]. Adenylyl cyclase (AC) is a membrane-bound enzyme that converts adenosine triphosphate (ATP) to cyclic adenosine monophosphate (cAMP) [14], which acts upon four effector proteins: EPAC, cyclic-nucleotide gated ion channels (CNGC) [15], Popeye proteins [16] and, more classically, cAMP-dependent PKA. PKA phosphorylates a vast number of substrates that transduce signals to many cellular locations. cAMP phosphodiesterases (PDEs) are a superfamily of enzymes which rapidly hydrolyse cAMP to adenosine 5′-monophosphate (5′-AMP), and therefore inactivation of PDEs increases the amount of intercellular cAMP [17]. PDEs are localised in cells to maintain the fidelity of cAMP signals spatially and temporally [18].

PKA exists as a tetrameric holoenzyme consisting of a regulatory (R) subunit dimer and two catalytic (C) subunits. When inactive, the PKA holoenzyme consists of dimerised regulatory subunits bound to two catalytic subunits. The regulatory subunits anchor the PKA holoenzyme to A-kinase anchoring proteins (AKAP) [19,20] which sequester PKA in specific cellular compartments. Binding of cAMP to the regulatory subunits results in activation of PKA via a conformational change where the active catalytic subunits and dimerised regulatory subunits dissociate. The catalytic subunits, which contain the active site and act as protein kinases, may then phosphorylate downstream targets. Phosphoinositide-dependent protein kinase 1 (PDK1) phosphorylation at Thr197 is also needed for full PKA activation [21]. Four regulatory subunits (RIα, RIβ, RIIα and RIIβ) and three catalytic subunits (Cα, Cβ and Cγ) of PKA have been identified [22]. The two major forms of PKA, known as PKA type I (PKA-I) and PKA type II (PKA-II) are comprised of different regulatory subunits interacting with identical catalytic subunits. PKA-I and PKA-II also differ in intercellular location, with PKA-I located in the cytosol and PKA-II located in plasma, nuclear and mitochondrial membranes and the particulate fraction [23]. In both PKA-I and PKA-II, once activated, the catalytic subunits translocate to the nucleus whilst the regulatory subunits remain in the cytosol. The function and physiology of the Cα subunit of PKA (encoded by the gene *PRKACA*) is reviewed by Turnham and Scott [24]. The cellular ratio of PKA-I to PKA-II differs between tissues and changes to this ratio have been implicated in cancer development, including prostate cancer [25,26].

## PKA in prostate cancer

In 2000, PKA-I was found to be overexpressed in various cancers, including prostate cancer [27], and, specifically, PKA-I RIα is constitutively overexpressed in prostate cancer [28]. Sustained activation of PKA is associated with prostate cancer progression [29]. Several studies have shown that by blocking or down-regulating PKA, prostate tumour growth can be slowed [30,31].

More recently, specific components of the PKA holoenzyme have been implicated in prostate cancer progression and response to therapies. Paclitaxel (Taxol) is a chemotherapy drug prescribed for several cancers and PKA/PKA holoenzyme components are known to regulate the response of prostate cancer cells to Paclitaxel treatment [32]. However, paclitaxel is not licensed for prostate cancer treatment. Following Taxol treatment, DNA- and RNA-based mapping showed that mutant cells with a randomly inserted strong promoter in the first intron of *PRKAR2A*, the gene coding for PKA-RIIα, survived both treatments, suggesting that *PRKAR2A* is a candidate Taxol-resistance gene. Further experiments on *PRKAR2A* gene function showed that overexpression of full-length or N-terminal truncated RIIα increased the number of colonies formed by prostate cancer cell lines. N-terminal truncated RIIα lacks the domain necessary for intercellular tethering and localisation (e.g. binding to AKAPs) but retains the domain necessary for interaction with PKA catalytic subunits leading to the hypothesis that Taxol resistance and increased prostate cancer cell survival occurs because the overexpressed RIIα subunit inhibits the activity of PKA catalytic subunits. In support of this, addition of a PKA inhibitor increased Taxol-treated prostate cancer cell survival [32]. However, no clinical benefits of Taxol treatment have been found in prostate cancer while the related chemotherapeutic, docetaxel (Taxotere), clinically benefits patients with metastatic hormone-sensitive prostate cancer and metastatic CRPC [33,34].

Another component of the PKA holoenzyme, RIIβ, has been suggested as a novel oncogene in prostate cancer. By screening the ONCOMINE database (a cancer microarray database), significant *PRKAR2B* overexpression was identified in both hormone-refractory prostate cancer (*P* < 0.001) compared with hormone-sensitive prostate cancer and metastatic tissue (*P* < 0.05) compared with primary site, suggesting that RIIβ contributes to CRPC [35]. RIIβ-siRNA knockdown reduced proliferation of CRPC cell lines DU145 and PC3, whilst RIIβ overexpression increased proliferation in the CRPC cell line 22Rv1 [35]. However, in the castration-sensitive cell line LNCaP, RIIβ-siRNA knockdown did not affect cell invasion, suggesting that the oncogenic role of RIIβ is limited to CRPC cells. Additionally, RIIβ-siRNA knockdown increased expression of the apoptosis marker cleaved PARP and sensitised DU145 cells to chemotherapy treatment with cisplatin, suggesting that RIIβ has an anti-apoptotic and pro-survival role in CRPC. Next, analysis of the whole genome transcriptome of RIIβ-siRNA knockdown in DU145 cells identified 385 significantly altered genes regulated by RIIβ, with the majority involved in cell cycle regulation and proliferation, highlighting the importance of RIIβ [35].

In a later study, Sha et al. [36] showed significant RIIβ overexpression in an androgen-independent LNCaP cell line (LNCaP-AI) (*P* < 0.05) compared with androgen-dependent LNCaP cell line as well as significant RIIβ overexpression in metastatic CRPC tissue (*n* = 17, *P* = 0.017) compared with primary prostate cancer tissue. Additionally, there was a significant relationship between RIIβ overexpression and higher Gleason score (*P* = 0.021) and lymph node metastases (*P* = 0.039), indicating that RIIβ may be linked to patient outcome. Strikingly, patients with high RIIβ expression (expression higher than median) had significantly shorter overall survival (*n* = 37, *P* = 0.031) than patients with low RIIβ expression. RIIβ overexpression promoted LNCaP cell invasion whilst RIIβ knockdown reduced LNCaP-AI cell invasion. Corresponding with the *in vitro* results, RIIβ overexpression increased tumour metastasis *in vivo* in a metastatic cancer mouse model. To further elucidate the role of RIIβ in metastasis, RIIβ overexpression in LNCaP-AI cells was shown to activate Wnt/β-catenin signalling and promote epithelial-to-mesenchymal transition (EMT) [36]. Taken together, RIIβ may be a potential therapeutic target in the treatment of metastatic prostate cancer.

Another study from the same group found that RIIβ overexpression in prostate cancer is not a consequence of gene mutations, copy number changes or epigenetic modifications such as methylation and histone acetylation [37]. Subsequently, RIIβ overexpression was hypothesised to be the result of micro-RNA (miRNA) gene regulation at the post-transcriptional level, and indeed, two miRNAs were identified as upstream regulators of RIIβ expression in prostate cancer cell lines [38]. These miRNAs were then shown to be significantly down-regulated in metastatic CRPC (*P* < 0.01) and their expression level was negatively associated with RIIβ expression, although this correlation was weak (*R*^2^ = 0.22, 0.45 for the two miRNAs). Binding sites for several transcription factors were identified in the *PRKAR2B* promoter from JASPAR and TRANSFAC database searches, and of these transcription factors, X-box binding protein 1 (XBP1) knockdown was shown to inhibit RIIβ expression. miRNA expression or XBP1 knockdown reduced cell viability and increased apoptosis in prostate cancer cell lines, whilst overexpression of RIIβ prevented these tumour suppressive effects. In summary, regulators of RIIβ expression are another potential therapeutic target for prostate cancer treatment.

The Warburg effect describes the increase in glucose uptake and lactate production as part of metabolic reprogramming undertaken by cancer cells to promote growth, survival and proliferation. In a recent paper, RIIβ was shown to regulate the Warburg effect in prostate cancer by up-regulating hypoxia-inducible factor 1 (HIF-1), a transcription factor that mediates the Warburg effect [38]. Subsequently, RIIβ expression was found to be induced under hypoxic conditions and, as HIF-1α is the mediator of the response to hypoxic stress, a positive regulatory feedback loop involving RIIβ and HIF-1α was identified in prostate cancer. Finally, in an *in vivo* subcutaneous xenograft model of prostate cancer, the pro-tumour effects of RIIβ were shown to be glycolysis dependent. This data provides additional targets for prostate cancer therapeutic development such as targeting the RIIβ/HIF-1α feedback loop.

## The androgen receptor

The AR is a nuclear receptor activated by androgens, a group of hormones that includes testosterone and dihydrotestosterone (DHT). In its ligand-unbound and inactive state, the AR is stabilised in the cytoplasm by heat shock proteins (HSPs), co-chaperones and other proteins in a large complex [39]. The AR has two mechanisms of action. The first is a genomic mechanism of action: ligand binding leads to dissociation of the AR from the stabilisation complex and AR translocation into the nucleus where it dimerises and binds androgen response elements (AREs), regulating transcription of androgen-targeted genes [40]. The second is a non-genomic mechanism of action where the AR can have interactions independent of those with DNA [41,42]. For example, the AR can interact with signal transduction proteins in the cytoplasm, indirectly regulating the expression of genes in multiple other signalling pathways. One specific example of this gene regulation of signalling pathways is the cross-talk between the AR and the cAMP-dependent PKA signalling pathway, which will be discussed here. As an aside, Foley and Mitsiades [43] provide a comprehensive review on AR-interacting proteins in prostate cancer.

## AR in androgen-dependent and castration-resistant prostate cancers

Both normal prostate tissue and most early-stage prostate tumours are androgen-dependent for growth and proliferation. Early-stage prostate tumours are most often treated with radiation therapy and this may be in combination with ADT. Unfortunately, in many cases, ADT only provides a temporary reduction to prostate cancer growth and tumour progression and relapse into an aggressive castration-resistant tumour is common. Most castration-resistant tumours express a functional AR and therefore, the term androgen-independent prostate cancer has been replaced with CRPC. Continued AR activation under low levels of androgen following ADT has been observed in CRPC cells and suggested mechanisms behind this are reviewed by Nadiminty and Gao [44]. One mechanism that has been suggested is AR splice variants and their clinical relevance is reviewed by Wach et al. [45]. Additionally, it is thought that reactivation of the AR occurs via signalling pathways other than the androgen signalling pathway, such as the cAMP/PKA pathway, during the progression to CRPC [46,47]. There is evidence that the AR also regulates the EMT in prostate cancer and ADT has been shown to activate EMT and neuroendocrine differentiation (NED), which will be discussed later in this review [48,49].

Eder et al. [50,51] found that antisense silencing of AR expression (siAR) attenuated prostate tumour growth both *in vitro* and *in vivo*. Subsequently, this research group inhibited PKA activity by PKA-RIα-siRNA in both androgen-sensitive (LNCaP and VCaP) and androgen deprivation-resistant (LNCaPabl) prostate cancer cells [31]. Interestingly, AR-siRNA treatment reduced PKA-RIα expression in cells with functional AR and, correspondingly, PKA-RIα-siRNA treatment reduced AR expression, indicating a clear cross-talk between the AR and PKA pathways [31].

It is known that AR knockdown inhibits prostate cancer cell growth in both androgen-sensitive and androgen-insensitive cell lines [52]. Desiniotis et al. next investigated whether simultaneously knocking down AR and PKA-RIα would inhibit cell growth to a greater extent than AR knockdown alone. In androgen-sensitive LNCaP cells, proliferation decreased by 72–75% after double siRNA knockdown of AR and PKA-RIα, an improvement on single knockdown of AR or PKA-RIα by 40–48% and 25%, respectively [31]. The double knockdown of AR and PKA-RIα increased caspase-3 activity (associated with cancer cell apoptosis) by 17- or 18-fold, significantly more than the single knockdown (AR, 9- or 11-fold; PKA-RIα, 7.5-fold). Interestingly, in androgen-sensitive VCaP cells, the double knockdown was much less efficient and only a 2- or 2.5-fold increase in activity was seen, however, VCaP cells have significantly higher AR and PKA-RIα expression levels than the LNCaP cell line. This result clearly demonstrates that cAMP/PKA pathways cross-talk with AR signalling routes and directly regulate the expression of AR; this relationship appears to be dependent upon the androgen-sensitivity status of the cell line.

The standard prostate cancer treatment is anti-androgen therapy, or ADT. Bicalutamide is an anti-androgen which aims to block the activation of the AR. Desiniotis et al. [31] treated LNCaP cells with bicalutamide and siPKA, observing a reduction in proliferation of 60% (*P* < 0.001) compared with 25% reduction after bicalutamide treatment alone. In contrast, in the androgen deprivation-resistant LNCaPabl cell line, bicalutamide treatment increased proliferation by 23% and siPKA treatment did not decrease proliferation. Additionally, in LNCaP cells treatment of both bicalutamide and H-89 (a PKA inhibitor) decreased proliferation by 58%, whilst in LNCaPabl cells, this combination treatment had no effect on proliferation [31]. The LNCaPabl cell line may therefore reflect the transition from androgen-dependent to castration-resistant status that causes ADT resistance *in vivo*.

Following on from this work, the utility of combination therapy using oligodeoxynucleotides against AR and PKA-Riα was investigated [53]. Initial *in vitro* experiments showed that combined treatment with AR and PKA oligodeoxynucleotides decreased the androgen-sensitive LNCaP cell number to 24.6% of control, only a slight improvement on the single AR oligodeoxynucleotide treatment (33.6% of control, *P* = 0.041). Combination treatment with the AR and PKA oligodeoxynucleotides increased the number of apoptotic cells 3.5-fold over the control. Treatment with the PKA oligodeoxynucleotide alone decreased AR protein levels by 39.7%, confirming previous findings by this group that AR and PKA-RIα interact with each other. In androgen deprivation-resistant LNCaPabl cells, the oligodeoxynucleotides did not significantly inhibit growth.

Next, they looked to replicate these findings in an *in vivo* mouse model of prostate cancer [53]. Mice with LNCaP subcutaneous tumours treated with combination AR and PKA oligodeoxynucleotides over four weeks had significantly reduced tumour mass compared with control (*P* = 0.008) [53]. Excitingly, mice with LNCaPabl subcutaneous tumours treated with combination AR and PKA oligodeoxynucleotides showed complete tumour remission. These results indicate that targeting both AR and PKA signalling pathways by siRNAs or oligodeoxynucleotides inhibits prostate cancer growth in both *in vitro* and *in vivo* settings. Taken together, these results suggest that PKA-RIα may be an appropriate biomarker for patients overexpressing PKA and who will respond well to PKA-RIα knockdown and ADT. However, the authors stated an assumption that targeting PKA-RIα in patients would result in regulation of PKA signalling; this would need to be investigated.

## cAMP/PKA can regulate AR activation in prostate cancers

It has been known since the 1990s that increased cAMP activates the AR, however, this occurs in both androgen-dependent and androgen-independent ways. In their original review, Merkle and Hoffmann summarised several papers which reached contrasting conclusions: that forskolin, an activator of AC and a PKA stimulator, activates the AR independent of androgens; and that forskolin and androgens co-activate the AR [29]. Contemporary research in this area is summarised here.

**Figure 1.**
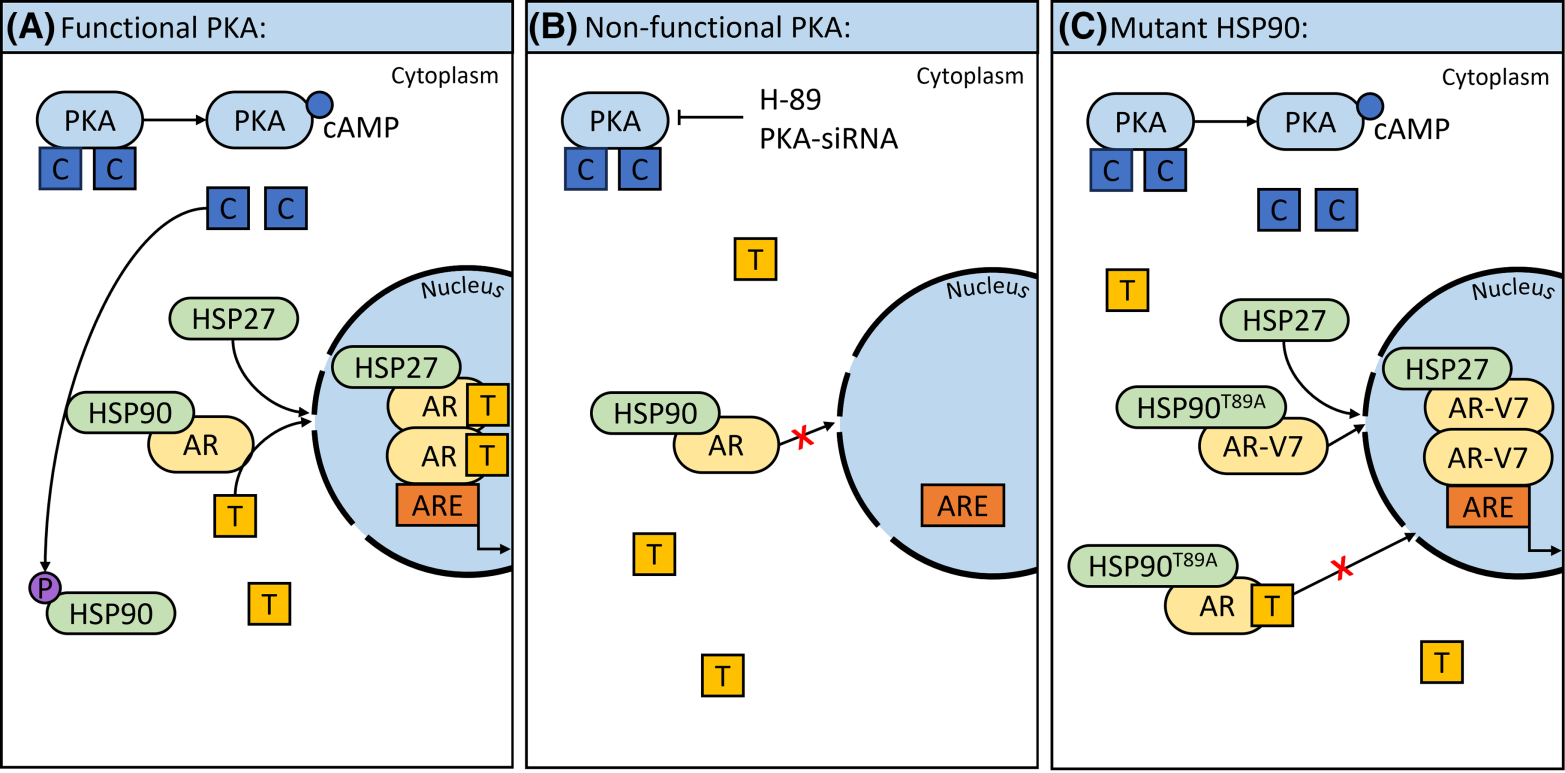
cAMP and PKA can regulate AR activation and translocation. (**A**) cAMP binds to PKA resulting in a conformational change where its catalytic subunits dissociate. When ligand is not bound to AR, it is stabilised in the cytoplasm by proteins including HSP90. The catalytic subunits of PKA phosphorylate HSP90, freeing the AR which binds to its ligand, androgen, as well as HSP27 and translocates to the nucleus. The AR then binds to the ARE resulting in transcription of target genes. (**B**) Treatment with either H-89 or PKA-siRNA prevents activation of PKA and so translocation of AR to the nucleus is blocked and transcription of target genes prevented. (**C**) Mutant HSP90^T89A^ which is unable to be phosphorylated by PKA, prevents nuclear translocation of AR even when androgen is bound. However, nuclear translocation of splice variant AR-V7 occurs in presence of mutant HSP90T89A and absence of ligand binding. (AR = androgen receptor, AR-V7 = androgen receptor splice variant, ARE = androgen response element, C = catalytic subunit of PKA, cAMP = cyclic adenosine monophosphate, HSP = heat shock protein, P = phosphoryl group, PKA = protein kinase A, siRNA = small interfering RNA, T = androgen). *Parts of the figure were drawn by using pictures from Servier Medical Art. Servier Medical Art by Servier is licensed under a Creative Commons Attribution 3.0 Unported License (*https://creativecommons.org/licenses/by/3.0/*)*.

A key part of AR activation involves its translocation to the nucleus [54] and thus Dagar et al. [55] investigated the importance of PKA activation in AR nuclear translocation [56] (Figure 1). The ligand unbound form of AR is localised in the cytoplasm and stabilised in a complex by molecular chaperones such as HSP90; once bound to androgen, the AR dissociates from HSP90 and associates with HSP27 [57]. In agreement with the literature, LNCaP cells treated with androgen showed time-dependent nuclear localisation of GFP-tagged AR whilst HSP90 remained in the cytoplasm [55]. However, AR nuclear localisation was impaired in androgen-treated LNCaP cells that had undergone H-89 treatment or siRNA-mediated PKA silencing. These results together indicate that PKA activity is necessary for AR dissociation from HSP90 and nuclear translocation. It was then demonstrated that following androgen binding, phosphorylation of HSP90 at Thr^89^ by PKA was required for AR release, association with HSP27 and nuclear translocation. Clinically, AR variants such as AR-V7 and AR^v567es^ have been found which lack the ligand binding domain yet still translocate to the nucleus and transcribe target genes independent of androgen, driving castrate-resistant growth. Overexpression of HSP90 T89A mutant significantly reduced transcription of WT AR with no effect on AR-V7 or AR^v567es^ transcription, indicating that HSP90 is not required for androgen-independent transcription. In summary, this study further elucidated the cross-talk between PKA, HSP90 and AR in prostate cancer [55].

A recent study performed a proteomic analysis of androgen treated or forskolin-treated VCaP cells (a model CRPC cell line) and identified eight proteins with significant expression changes compared with untreated cells [58]. Expression of three of these proteins was increased by androgen-induced signalling whilst five were increased by forskolin treatment, i.e. androgen-independent signalling, and likely PKA signalling. Interestingly, most of the proteins identified are involved in metabolism and as we have described previously, metabolic reprogramming is known to occur as prostate cancer progresses towards CRPC. Further work is needed to analyse if these proteins relate to changes in drug response as the disease progresses.

## PKA activation in prostate cancer

Previously, the focus has been on how cAMP and PKA stimulation can affect AR activation. PKA activation is required for AR translocation to the nucleus and this translocation can be regulated by testosterone in androgen-sensitive cells. However, during the androgen-insensitive stage of prostate cancer, G protein-coupled receptors (GPCRs) are up-regulated, and it is mainly the G_s_ and G_i_ groups of G proteins that are involved in regulation of ACs, which synthesise cAMP and activate PKA. Thus, it is thought that aberrant activation of G proteins could result in activation of PKA and AR nuclear translocation in the absence of, or low concentration of, androgens.

A novel androgen-binding GPCR, GPR56, was identified by BLAST analysis of 826 mammalian GPCRs as having high similarity to the ligand binding domain of the AR [59]. GPR56 was then shown to be expressed in several prostate cancer cell lines and overexpressed in prostate tumour samples. Interestingly, overexpression of GPR56 increased cell proliferation in cell lines. Additionally, testosterone was shown to bind to the C-terminal of GPR56 and this was subsequently shown to stimulate AR activity in a concentration dependent manner in LNCaP, PC3 and HEK293 cells overexpressing GPR56. Interestingly, the increase in AR activity was not seen in cells treated with PKA-siRNA or GPR56-siRNA. Additionally, GPR56-siRNA significantly reduced expression of the AR target genes PSA and TMPRSS2. GPR56 was also shown to regulate nuclear translocation of AR and GPR56-siRNA prevented full nuclear translocation in a timely fashion (24 h vs 1 h). The next phase of investigation was to evaluate the effect of androgens upon this signalling cascade. In LNCaP cells expressing GPR56, testosterone treatment resulted in three times as great PKA activation whilst GPR56-siRNA reduced this activation to only 1.5 times control. Concurrently, cells expressing GPR56 significantly accumulated cAMP (*P* < 0.001). Finally, they investigated whether GPR56 activation via testosterone treatment activated the Rho signalling pathway, as is the case in other G proteins, and showed that both PKA and Rho signalling pathways are activated and that for Rho activation, PKA activation is required [59]. In summary, testosterone treatment activates a novel GPCR, GPR56, that is involved in AR signalling in prostate cells. Future work may involve elucidating if GPR56 may be activated in a low androgen environment such as in CRPC and it remains to be seen if GPR56 is a potential drug target for treatment of prostate cancer.

## Androgen and AR regulation of the β_2_-adrenergic receptor and PKA

**Figure 2.**
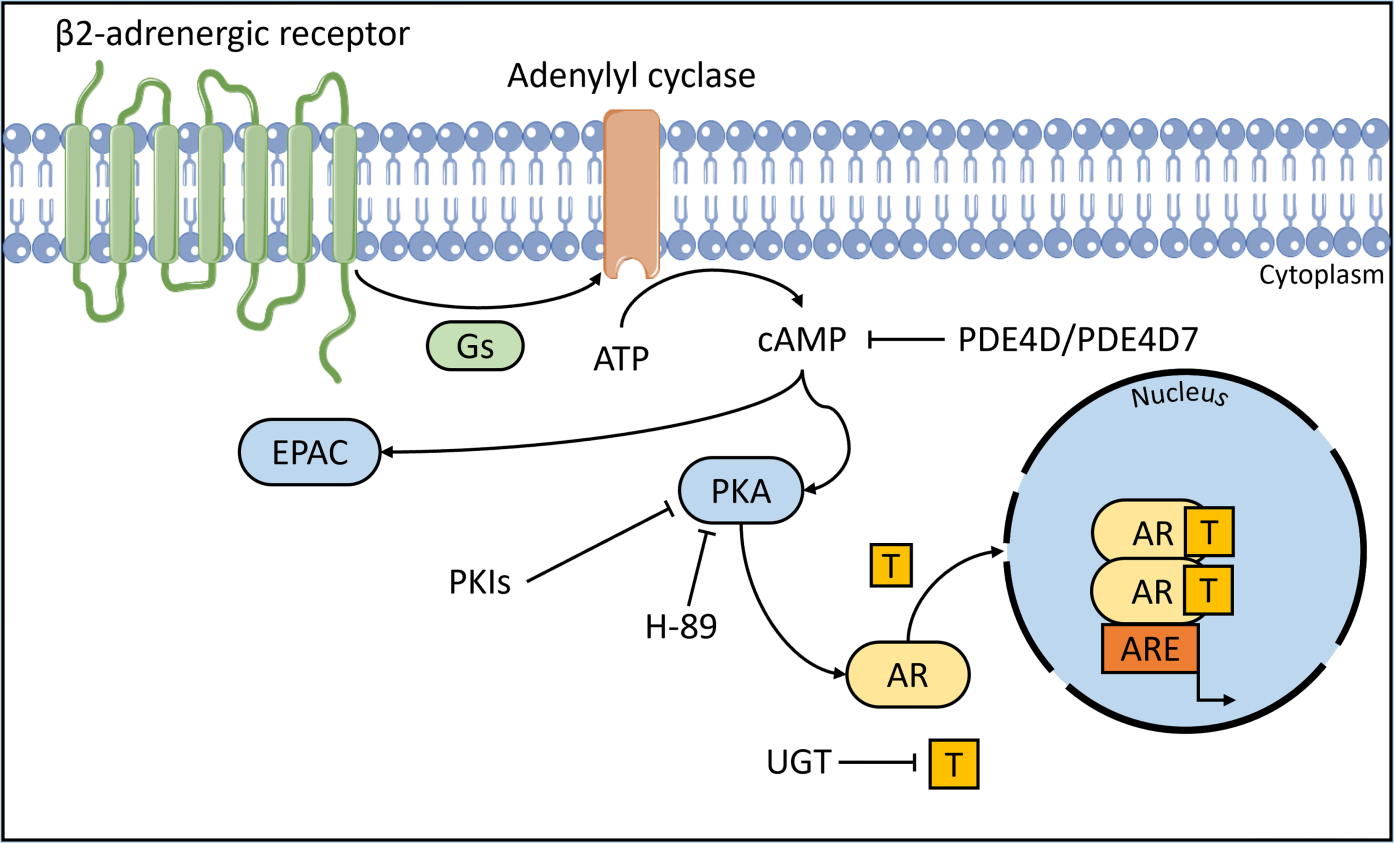
β2-adrenergic receptor signalling pathway. Adenylyl cyclase is stimulated by stimulatory G proteins following stimulation of the β2-adrenergic receptor. This increases intracellular cAMP levels which activates EPAC and PKA and, in the presence of androgens, results in translocation of AR to the nucleus and transcription of target genes. UGT enzymes control androgen inactivation. High levels of PKIs inhibit PKA signalling. (ATP = adenosine triphosphate, AR = androgen receptor, ARE = androgen response element, cAMP = cyclic adenosine monophosphate, EPAC = exchange protein directly activated by cAMP, Gs = stimulatory G protein, PDE4D = phosphodiesterase 4D, PKA = protein kinase A, PKIs = protein kinase A inhibitors, T = androgen, UGT = UDP-glucoronosyltransferases). *Parts of the figure were drawn by using pictures from Servier Medical Art. Servier Medical Art by Servier is licensed under a Creative Commons Attribution 3.0 Unported License (*https://creativecommons.org/licenses/by/3.0/*)*.

PKA is one of the β2-adrenergic receptor's (ADRB2) downstream cAMP-effector proteins and both cAMP and PKA are known to be involved in prostate cancer progression [60,61] (Figure 2). Androgen and AR signalling also play key roles in these signalling pathways and share several target genes. One research study examined whether the ADRB2 and its signalling pathways affected the progression of CRPC *in vivo* [62]. Immunohistochemical analysis of samples from a Norwegian patient cohort (*n* = 45) revealed that low levels of ADRB2 expression in tissue coincided with faster progressing CRPC [62]. This group also used ADRB2 null LNCaP cell lines to investigate the potential role of this receptor. They observed an increase in AC levels, indicating that reduced ADRB2 levels have a functional effect within prostate cells. ADRB2 KO LNCaP cells were injected into NOD-SCID mice, and the tumours were seen to grow faster in castrated mice. Gene expression profiling of the ADRB2 silenced LNCaP cells revealed that UDP-glucuronosyltransferase (UGT) 2B15 and 2B17 were down-regulated; these enzymes control androgen inactivation in prostate cancer [63]. Genetic ablation of ADRB2 reduced androgen glucuronidation activity (85–95%) and this improved androgen responsiveness *in vitro*. This data led to the hypothesis that reduced glucuronidation activity led to accumulation of glucuronidable androgens, resulting in increased androgen responsiveness. Testosterone was also found to be increased in the knockdown cell lines. Taken together, altering glucuronidation is another mechanism by which ADRB2 can regulate the AR in prostate cancer cells [62]. Further work from this group will be considered in a later section on NED.

Over a decade ago, data emerged showing that androgens alter the expression levels of PKA-R and PKA-C subunits in prostate cancer cells [64]. This study also showed that PKACβ2 is overexpressed in prostate tumour samples. Later work by the same group evaluated PKA subunits at the mRNA level in formalin-fixed paraffin-embedded (FFPE) prostate tissue from patients (*n* = 22) [65]. PKA Cβ2 mRNA was increased 1.7-fold in prostate cancer tissue compared with patient-matched benign tissue (*n* = 22). The same result was observed in a larger patient group (*n* = 169) where Cβ1 and Cβ2 were significantly higher in prostate tumour; expression level compared with benign ranged between 0.19 and 15.03, however all patients with Gleason score >7 (*n* = 6) had a mRNA ratio of <2 [65]. Cβ2 mRNA level is therefore negatively correlated with Gleason score, however there was no correlation between PSA level and Cβ2 mRNA level. As a high Gleason score indicates that progression is likely, low Cβ2 mRNA levels indicate reduced time until prostate cancer-specific mortality (PCSM). This was the first study to show a PKA C subunit correlating with PCSM. Pollack et al. [66] suggested that RIα can be a prognostic marker for patient outcome following radiation treatment. The results of Moen et al. [65] suggested that RIα can be combined with Cβ2 to create a prognostic marker that reflects the PKA holoenzyme. More research needs to be done investigating how the PKA holoenzyme changes during prostate cancer progression.

## Other factors affecting cAMP and PKA signalling in prostate cancer

### Protein kinase A inhibitor (PKI)

In addition to the points discussed above, there are other factors that may influence cAMP and PKA signalling in prostate cancer, and thereby affect cross-talk with the AR. The protein kinase A inhibitor (PKI) protein family is a group of three short proteins (PKIα, PKIβ and PKIγ). PKIs can specifically inhibit the kinase activity of PKA by binding to the catalytic subunits as pseudo-substrates and exporting the free catalytic subunit from the nucleus [67]. However, each member of the PKI family has a different affinity for PKA and distinctive intracellular localisations. By inhibiting PKA activity, it has been suggested that PKIs can regulate GPCR-cAMP activation. A review of PKI inhibition of PKA is provided by Liu et al. [68].

Merkle and Hoffmann [29] provided an excellent review of the work of Chung et al. [69] on this topic. Briefly, PKIβ was found to be overexpressed in androgen-insensitive prostate cancer cells, compared with normal prostate cells and androgen-sensitive prostate cancer cells. PKIβ overexpression resulted in an aggressive phenotype in prostate cancer cells. Finally, PKIβ was suggested to assist the nuclear import of the catalytic subunit of PKA or prevent nuclear export [69].

cAMP is known to signal through the MAPK/ERK pathway; this involves recruitment of Raf to either Ras or Rap1. PKA inhibits Ras signalling by phosphorylating either C-Raf on serine 259 or B-Raf on serine 365 [70,71]. Despite being unable to bind to Ras, phosphorylated B-Raf can still signal via Rap1 [70]. This group went on to suggest a model of cAMP-mediated ERK activation whereby PKA phosphorylation of B-Raf does X and PKA phosphorylation of Rap1 does Y [72].

In a recent study, expression levels of PKIs were shown to affect activation of both PKA and EPAC (a cAMP-effector that acts as a guanine exchange factor) [73]. The effects of PKI expression levels were obtained by observing ERK activation downstream of cAMP. Thus, PKIs are a molecular switch, preventing PKA signalling and activating EPAC signalling resulting in enhanced MAPK signalling. Additionally, an association was found between PKIα expression and an aggressive prostate cancer phenotype. siPKIs reduced PKIα mRNA in the CRPC cell line DU145 and this reduced migration *in vitro* and tumour growth potential *in vivo*. Conversely, PKIβ appeared to have no effects in DU145 cells [73].

### Phosphodiesterases

Other important signalling intermediates that are important in this area are PDEs. The PDE superfamily comprises eleven gene families (PDE1–PDE11), each containing numerous genes. PDE enzymes possess cyclic nucleotide-degrading activity and cAMP specific PDEs are negative regulators of the cAMP/PKA pathway. PDEs are known to be compartmentalised within the cell, allowing localised cAMP concentrations to be tightly regulated, with phosphodiesterase 4 (PDE4) family of particular relevance [17]. A review summarising PDE4 inhibitors in clinical trials may be found by Crocetti et al. [74]. Additionally, Mironid® Limited are developing novel PDE4 long-form selective inhibitors.

There is a negative correlation between AR and PDE4B in cancer [75]. In metastatic specimens (*n* = 25), PDE4B mRNA expression was significantly lower than that seen in control tissue. Decreased expression of this cAMP specific PDE may be a factor in the constitutively active AR pathway seen in metastatic prostate cancer. Additionally, the mRNA for another negative regulator of cAMP signalling, RIα, was lower in primary and metastatic cancer than in normal prostate tissue, while R2β mRNA was higher in metastatic compared with primary or normal tissue. R2β mRNA expression correlated with AR expression [75]. Other work evaluating PDE4B isoforms in prostate cancer, observed that PDE4B was down-regulated and the PKA signalling pathway was activated in a castration-resistant LNCaP cell line [76]. This PDE4B down-regulation appeared to promote cell proliferation [76]. Activation of the PKA signalling pathway led to AR activation, as seen by LNCaP cells with down-regulated PDE4B growing in androgen-deprived conditions**.**

Sarwar et al. [75] showed that cAMP-dependent/PKA pathways regulate AR and AR-dependent PSA expression in prostate cancer, independent of androgens. Rolipram is a selective PDE4 inhibitor that increases intracellular cAMP concentration in cells and PKA activity as well as nuclear AR activation and PSA expression [29,77]. They found that in the absence of androgen, rolipram-treated LNCaP cells showed increased AR and PSA expression, whilst forskolin-treated (a PKA stimulator that increases cAMP activity) LNCaP cells only showed increased PSA expression [75]. Interestingly, combined rolipram and forskolin treatment did not change AR or PSA expression, indicating that rolipram and forskolin must act via different mechanisms [75]. Since androgen treatment is known to increase AR and PSA expression, they subsequently showed that combined androgen, rolipram and forskolin treatment significantly increased expression of PSA (*P* < 0.01) and PKA subunits RIα, RIIβ and Cα in LNCaP cells. Forskolin-treated androgen-sensitive LNCaP cells showed increased RIIβ and Cα expression and no change in RIα expression. However, in androgen-insensitive PC3 cells, which do not express AR or PSA, forskolin treatment completely removed RIα expression, reduced Cα expression and slightly increased RIIβ expression. Since the cAMP/PKA pathway differs between androgen-sensitive and -insensitive cells, this pathway is functionally associated with androgen status.

PDE4D isoforms are also down-regulated in androgen-insensitive prostate cancer cell lines, with PDE4D7 being of particular importance in regulating prostate cancer proliferation and suggested as a novel tumour biomarker [78]. mRNA levels of PDE4 families and specific isoforms were analysed in 19 prostate cancer cell lines and xenografts and total PDE4D mRNA was found to decrease during the transition to androgen-insensitivity [78]. This was found to be mostly due to decrease in PDE4D7 expression at both the mRNA and protein levels. cAMP levels are regulated by the localisation of a population of PDE4D7 to the plasma membrane and interestingly, disruption of PDE4D7 localisation by siPDE4D7 led to increased cell proliferation above the level of siPDE4D. This indicates that PDE4D7 has a particularly important role in prostate cancer. Finally, PDE4D7 regulation was found to be independent of the canonical androgen signalling regulatory pathway.

Next, regulation of the long-form PDE4, PDE4D7, was investigated in more detail. Long-form PDE4s, contain two conserved regulatory domains (UCR1 and UCR2) which can be phosphorylated by PKA when cAMP concentrations are high resulting in PDE4 activation and hydrolysis of cAMP. Peptide array confirmed a second site (serine 42) in the unique N-terminal region of PDE4D7 that may also be phosphorylated by PKA [79]. This was confirmed by western blotting of HEK293 S42 mutants and wild-type cell treatment with forskolin increased phosphorylation indicating that PKA was responsible. Since PDE4D7 activity had previously been found to be down-regulated during the transition to androgen-insensitivity [78], Byrne et al. [79] found that S42 phosphorylation occurred in two androgen-sensitive cell lines DuCaP and VCaP. The effect of S42 phosphorylation on PDE4D7 activity was then investigated with a phosphorylation-resistant mutant being >170% more active than wild-type as well as resistant to forskolin treatment. Interestingly this mutant also had very little phosphorylation of UCR1, and it was suggested that S42 phosphorylation results in hyperactivity of PDE4D7, reducing cAMP levels and this prevents PKA from phosphorylating the UCR1.

The clinical relevance of PDE4D7 down-regulation was investigated by RNAseq analysis of 1405 tumour samples. PDE4D7 was found to initially increase in early-stage prostate cancer followed by down-regulation in CRPC [80]. PDE4D7 expression correlated with *TPMRSS2-ERG* gene fusion, a common gene fusion in prostate cancer with functionality yet to be fully determined. Most interestingly, PDE4D7 expression was found to correlate with disease aggression with low PDE4D7 expression correlating with poorer clinical outcomes and clearly implicating PDE4D7 as a potential biomarker of prostate cancer.

Next, the expression of other PDE4D isoforms in prostate cancer was investigated due to their ability to regulate downstream signalling pathways [81]. Two long PDE4D isoforms, PDE4D5 and PDE4D9, were found to be down-regulated in primary prostate cancer with expression continuing to decrease as the disease progresses to CRPC. Interestingly, metastatic samples showed heterogeneity in PDE4D isoform expression. Subsequently, they considered ERG regulation of PDE4D isoforms and found that ERG positive samples showed PDE4D7 overexpression with no effect on PDE4D1/2 and PDE4D9 and a potential weak effect on PDE4D5 expression. PDE4D isoform expression was investigated in LNCaP cells grown in androgen-free media and treated with the synthetic androgen R1881. PDE4D9 expression was unaffected whilst PDE4D5 expression was inhibited and PDE4D7 expression was up-regulated. Next, they identified hypermethylation sites on PDE4D5 in prostate cancer, indicating silencing by DNA methylation of promoters. Using surgical resections and needle biopsies from patients they measured PDE4D5 and PDE4D7 expression by qPCR and found inverse correlation with PDE4D5 expression decreasing close to the tumour and PDE4D7 expression increasing. Building upon their previous work, they created a prognostic score based on expression of PDE4D1/2 relative to the total expression of PDE4D5, PDE4D7 and PDE4D9. Using existing datasets this score was able to predict patients with clinical recurrence from those without.

A subsequent study investigated the association between PDE4D7 expression and clinical outcome in a prostate cancer cohort who had undergone resection (*n* = 503) [82]. PDE4D7 expression was analysed using quantitative real-time PCR (RT-qPCR) and transformed to a PDE4D7 score ranging 1–5. The Cancer of the Prostate Risk Assessment (CAPRA) score was calculated for this cohort as described by Cooperberg, Hilton and Carroll [83]. PDE4D7 score significantly correlated to time to postsurgical biochemical recurrence (*P* < 0.0001) with those patients in the highest PDE4D7 score category having <5% probability of recurrence within five years. The PDE4D7 score was combined with the CAPRA score and this new score was a better predictor of survival post-surgery. The potential implications of this score would be to identify those patients who may be at greater risk of recurrence post-surgery, and this could inform treatment plans for high-risk patients.

Next, the PDE4D7 and CAPRA scores were assessed for prognostic ability to stratify patients in three patient cohorts (radical prostatectomy cohort, *n* = 550; surgical cohort, *n* = 130; presurgical needle biopsy cohort, *n* = 151) [84]. The PDE4D7 score was significantly associated with time to biochemical relapse in both the radical prostatectomy (*P* < 0.0001, hazard ratio (HR) = 0.53 (0.41–0.67)) and presurgical needle biopsy cohort (*P* < 0.0001, HR = 0.47 (0.33–0.65)). When the PDE4D7 score was combined with the CAPRA score in a sub-cohort of the radical prostatectomy cohort (*n* = 449), the combined score was able to predict 5-year risk of biochemical relapse after surgery (*P* = 0.0002, odds ratio = 0.46 (0.3–0.69)). Additionally, combined PDE4D7 and CAPRA score's HR was increased to 16.4 compared with the CAPRA score alone (HR = 11.8) in the radical prostatectomy cohort. The prognostic power of combining PDE4D7 score with CAPRA score is promising, and future work in another patient cohort will elucidate whether this is in fact a prognostic biomarker.

Building upon this work, other long isoforms of PDE4D (PDE4D5 and PDE4D9) were considered for their prognostic ability in addition to PDE4D7 and as well as relating to TMPRSS2–ERG fusion status [85]. RT-qPCR determined expression of PDE4D5, PDE4D7 and PDE4D9 and RNA sequencing identified TMPRSS2–ERG fusion status. In those patients with TMPRSS2–ERG fusion, high PDE4D7 expression was associated with lowest risk of disease progression, using biochemical relapse as a surrogate endpoint. However, in those without the gene fusion, there was no significant difference between PDE4D7 categories and progression-free survival (*P* = 0.08). Interestingly, in those without the gene fusion, PDE4D5 score and PDE4D9 score both significantly associated with biochemical relapse (*P* < 0.0001 and *P* < 0.0001, respectively) whilst in those with the gene fusion present, PDE4D9 score significantly associated with biochemical relapse (*P* = 0.005) and PDE4D5 was not significantly associated. Additionally, they found that reduced expression of multiple long PDE4D isoforms increased the risk of developing metastases or dying from prostate cancer. Next, they developed a combined model comprising the CAPRA score with the scores for PDE4D5, PDE4D7 and PDE4D9 expression. The combined model stratified patients into a group with no risk of postsurgical disease progression (*n* = 36, 23.8%). Additionally, those patients with the highest scores in the combined model had the highest risk of biochemical recurrence within five years of 63.9% (score 3–4) and 83.3% (score 4–5). When compared with the previous CAPRA score model, the combined PDE4D5/7/9 and CAPRA score model increased the area under the curve by 10%, indicating that including PDE4D5 and PDE4D9 increases the prognostic power of the CAPRA PDE4D7 score. The combined PDE4D5/7/9 and CAPRA score was subsequently shown to be associated with elevated Gleason grade (*P* < 0.0001) and extended tumour growth after resection [86]. A group of low-risk patients were identified by applying a cut-off to the combined score and none showed disease progression during >7.5 years of follow-up, indicating that these patients may be suitable for active surveillance, reducing unnecessary primary treatment. Taken together, these studies indicate that the CAPRA and PDE4D5/7/9 score is suitable to identify both high and low-risk patients and could be useful to inform treatment strategies.

### Exchange protein directly activated by cAMP

The cAMP-responsive guanine nucleotide exchange factor (cAMP-GEF) is also known as EPAC and is an increasingly recognised cAMP-effector. EPAC has a cAMP binding domain similar to the regulatory subunits of PKA. There are two known isoforms called EPAC1 and EPAC2 which share sequence and structure, with the major difference being the N-terminal cAMP binding domains. Sugawara et al. [87] provided a comprehensive review of the structure of EPAC2 and an overview of EPAC in cancer may be found by Wehbe et al. [88]. EPAC acts upon numerous signalling pathways by activating both ERK1/2 and PI3K/Akt signalling pathways and it has also been suggested that EPAC may have an additional pro-inflammatory role in prostate cancer.

Misra and Pizzo [89] showed that EPAC stimulation by cAMP analogues increased prostate cancer cell proliferation. This outcome was a result of EPAC-induced activation of the B-Raf/ERK and mTOR signalling cascades. These signalling cascades act independently of PKA yet require cAMP. Previous work by this group also showed that in endothelial cells, EPAC activation by treatment with a selective EPAC agonist 8-CPT-2Me-cAMP (8CPT), stimulated Rap1 suppressing chemotaxis and angiogenesis. They then went on to look at this in a prostate tumour xenograft, finding that 8CPT treatment had no effect on tumour growth but in cells with constitutively active Rap1, 8CPT treatment inhibited tumour growth and VEGF expression and angiogenesis [90]. Further analysis showed that 8CPT stimulated PKA, not EPAC/Rap1 as in endothelial cells. It is therefore important not to immediately apply findings in endothelial cells to prostate cells, as clearly prostate cells preferentially activate PKA over EPAC. Future work on the role of PKA/cAMP/EPAC on the tumour and immune microenvironment in prostate cancer is necessary and would provide additional information on disease progression and effectiveness of treatment options. Next, EPAC was investigated in the context of a multiprotein complex. Co-immunoprecipitation showed that after 8CPT EPAC1 signalling, EPAC1 co-immunoprecipitated with PDE3B and PDE4D amongst other signalling proteins [91]. PDEs may therefore act as scaffolding proteins, localising EPAC1 binding and activity to designated compartments within the cell. EPAC1 promotes prostate cell proliferation and survival by up-regulating Ras-MAPK and PI3-kinase-Akt-mTOR signalling [92]. It remains to be seen whether EPAC is a suitable cancer therapy target for prostate cancer.

The conformational states of EPAC1 have been solved by Small-Angle X-ray Scattering (SAXS), revealing that the inactive conformation is a mixture of two forms, where 86% is the folded and compact form and 13% is an extended form [93]. This study suggested a novel intermediate conformation of EPAC1 in an inactive state. EPAC1 is in its active state when bound to cAMP and its effector Rap1b. EPAC1 and EPAC2 are expressed in all prostate cells of patients undergoing radical prostatectomy and expression levels appear to vary along with PSA [94]. EPAC expression appears to be colocalised with αSMA, a marker of smooth muscle cells. Stimulation of prostate tissues with EPAC activators activated the transcription factor Elk1, a novel finding and important given that in prostate cancer cells Elk1 affects proliferation. A review of EPAC activators may be found in a paper by Luchowska-Stańska et al. [95].

VPAC1 and VPAC2 receptors, class II GPCRs, bind and control effects of vasoactive intestinal peptide (VIP), mainly by increasing cAMP via Gs signalling. In prostate cell lines, the localisation of these receptors was unique with VPAC1 on the nuclear membrane and VPAC2 on the plasma membrane and in the cytosol [96]. VIP treatment in LNCaP and PC3 cells induced a twenty-five times higher level of cAMP compared with the histologically normal prostate cell line RWPE-1 and significantly increased nuclear translocation of p50, a subunit of NF-κB. Of interest was the finding that in RWPE-1 cells, the only signalling pathway involved in VIP effects is the PKA pathway, whilst in LNCaP cells the signalling pathway is cAMP/EPAC/PI3K and in PC3 cells numerous signalling pathways are used: cAMP/PKA, EPAC/ERK and PI3K pathways. As PC3 cells are androgen-insensitive, it is interesting that the signalling pathways used are so different from androgen-dependent LNCaP cells, suggesting an opportunity for differentially targeting the EPAC signalling pathway for late-stage prostate tumours. More recently, it has been suggested that in cancer stem cells, VIP signalling may be a method by which cancer stem cells are resistant to therapy [97]. A study in breast cancer showed promising results when they generated VIP-bound Paclitaxel, targeting the therapy to the VIP receptors overexpressed on breast cancer cells; it is possible a similar route may be useful in prostate cancer [98].

## Neuroendocrine cell differentiation

Prostate adenocarcinoma may transdifferentiate via NED into neuroendocrine prostate cancer (NEPC), a more aggressive and advanced tumour, induced by treatments including ADT or other therapies targeting components of the AR signalling pathway such as ADRB2 activation [99]. NEPC cells have altered morphology and are positive for several neuroendocrine markers, while having extremely low levels of AR and low AR-regulated gene transcription. NED is one method by which tumour heterogeneity may arise, a challenge for effective treatment, particularly conventional chemotherapies.

LNCaP cells are commonly used as a model of NED as they differentiate to a NE phenotype under certain conditions. One study investigated how cAMP elevation regulates initial stages of NED using LNCaP cells. They found that activation of PKA following increased cAMP levels promoted a NE phenotype, as seen by morphology changes with cells having protrusions and branching [100]. Next, they considered whether RhoA, a small GTPase in Rho family and a downstream substrate of PKA, played a role in NED. They inhibited RhoA signalling or inhibited Rho kinase and in both cases found significantly reduced length of cellular protrusions. It is known that PKA phosphorylates RhoA at Ser188, so they next transfected LNCaP cells with RhoA Ser188 mutants and treated with forskolin, an activator of membrane ACs, showing that mutants had reduced protrusion length and that RhoA phosphorylation is needed for changes in morphology. Taken together, their data showed that increased cAMP and PKA phosphorylation and inactivation of RhoA causes changes to cell morphology [100].

Further investigation into NED in LNCaP cell assessed levels of known markers of NED, γ-enolase, cytoskeletal protein tubulin β-III and aromatic L-amino acid decarboxylase, an enzyme known to interact with the AR [101]. Androgen-deprived LNCaP cells displayed increased levels of γ-enolase and tubulin β-III protein, alongside increased mRNA levels of γ-enolase and L-amino acid decarboxylase by qRT-PCR [101]. Similar up-regulation of NED markers was observed in LNCaP cells grown to high density with androgens present in the media. Analysis of this kind can inform the discussion of whether NED is reversible in prostate cancer cells. For cells that have undergone NED at high cell density, re-seeding at low density resulted in decreased γ-enolase expression, suggesting reversal of NED phenotype. However, for cells that have undergone NED because of androgen depletion, when added back into media containing FBS, γ-enolase levels remained high for up to 8 days, indicating that NED is not reversible under these conditions. Additionally, increased NED occurs in AR-positive and AR-negative prostate epithelial cell lines when grown to high density, dual inhibition of Cdk1 and Cdk2 modulated cell cycle and promoted NED and the cAMP-mediated pathway is activated under high-density conditions [101].

As described previously, the ADRB2 is involved in progression to CRPC [62]. This group went on to investigate whether ADRB2 is implicated in NED [102]. Using shRNAs targeting ADRB2 mRNA in LNCaP cells, they identified 51 differentially expressed genes with the most enriched pathways being neuron differentiation (*P* = 0.026) and neuron projection development (*P* = 0.028), pointing towards NED development. When LNCaP cells were treated with shADRB2 and cultured in androgen-depleted medium, they found that mRNA and protein levels of neuronal markers were decreased and cell projection length was significantly shorter, indicating that LNCaP shADRB2 cells were undergoing NED to a lesser extent. Treatment with forskolin, activating AC, removed this reduced projection length effect indicating that cAMP elevation causes NED, bypassing ADRB2. ADRB2 overexpression was shown to induce the morphology changes in both AR-positive and AR-negative cell lines, however there was no change in levels of neuroendocrine markers TUBB3, CHGA and SYP. In summary, ADRB2 promotes cell projection growth but has no effect on neuroendocrine markers. Interestingly, they found that in metastatic CRPC patient samples with NED histology, ADRB2 levels were significantly down-regulated compared with CRPC and low ADRB2 expression was associated with up-regulation of NE markers. This data agrees with the molecular work previously done and the group proposes that high ADRB2 expression may mean those cells are more prone to undergo NED following ADT.

## Conclusions

In updating the combined knowledge on the roles that PKA, cAMP and EPAC play in the development and progression of prostate cancer (specifically with respect to its cross-talk with AR signalling) this review positions the cAMP signalling system as a key future area for development in the design of modern and targeted therapies for this disease.

## Open Access

Open access for this article was enabled by the participation of University of Glasgow in an all-inclusive *Read & Publish* agreement with Portland Press and the Biochemical Society under a transformative agreement with JISC.

## Abbreviations

AC: adenylyl cyclase
ADRB2: β2-adrenergic receptor
ADT: androgen deprivation therapies
AR: androgen receptor
AREs: androgen response elements
CAPRA: Cancer of the Prostate Risk Assessment
CRPC: castration-resistant prostate cancer
EMT: epithelial-to-mesenchymal transition
EPAC: exchange protein directly activated by cAMP
HIF-1: hypoxia-inducible factor 1
HR: hazard ratio
NED: neuroendocrine differentiation
NEPC: neuroendocrine prostate cancer
PCSM: prostate cancer-specific mortality
PDE4: phosphodiesterase 4
PDEs: phosphodiesterases
PKA: protein kinase A
PKI: protein kinase A inhibitor
PSA: prostate-specific antigen
UGT: UDP-glucuronosyltransferase
VIP: vasoactive intestinal peptide
XBP1: X-box binding protein 1
